# Nucleoredoxin Redox Interactions Are Sensitized by Aging and Potentiated by Chronic Alcohol Consumption in the Mouse Liver

**DOI:** 10.3390/antiox13030257

**Published:** 2024-02-20

**Authors:** Osiris Germán Idelfonso-García, Brisa Rodope Alarcón-Sánchez, Dafne Guerrero-Escalera, Norma Arely López-Hernández, José Luis Pérez-Hernández, Ruth Pacheco-Rivera, Jesús Serrano-Luna, Osbaldo Resendis-Antonio, Erick Andrés Muciño-Olmos, Diana Ivette Aparicio-Bautista, Gustavo Basurto-Islas, Rafael Baltiérrez-Hoyos, Verónica Rocío Vásquez-Garzón, Saúl Villa-Treviño, Pablo Muriel, Héctor Serrano, Julio Isael Pérez-Carreón, Jaime Arellanes-Robledo

**Affiliations:** 1 Laboratory of Liver Diseases, National Institute of Genomic Medicine—INMEGEN, Mexico City 14610, Mexico; brisa.alarcon@cinvestav.mx (B.R.A.-S.); dguerrero@inmegen.gob.mx (D.G.-E.); nalopez@inmegen.gob.mx (N.A.L.-H.); jiperez@inmegen.gob.mx (J.I.P.-C.); 2 Department of Health Sciences, Div CBS, Metropolitan Autonomous University-Iztapalapa Campus, Mexico City 09340, Mexico; hser@xanum.uam.mx; 3 Department of Cell Biology, Center for Research and Advanced Studies of the National Polytechnic Institute—CINVESTAV-IPN, Mexico City 07360, Mexico; jesus.serrano@cinvestav.mx (J.S.-L.); svilla@cell.cinvestav.mx (S.V.-T.); 4 Department of Gastroenterology and Hepatology, Hospital General de México “Dr. Eduardo Liceaga”, Mexico City 06720, Mexico; jose.luis.perezhe@pemex.com; 5 Laboratory of Molecular Diagnostics, Department of Biochemistry, National School of Biological Sciences of the National Polytechnic Institute, Mexico City 07738, Mexico; rpachecor@ipn.mx; 6 Laboratory of Human Systems Biology, National Institute of Genomic Medicine—INMEGEN, Mexico City 14610, Mexico; oresendis@inmegen.gob.mx; 7 Coordination of Scientific Research—CIC, Research Support Network—RAI, Center for Complexity Sciences—C3, National Autonomous University of Mexico—UNAM, Mexico City 04510, Mexico; 8 Division of Translational Cancer Research, Department of Laboratory Medicine, Lund University, 22381 Lund, Sweden; erick.mucino_olmos@med.lu.se; 9 Laboratory of Genomics of Bone Metabolism, National Institute of Genomic Medicine—INMEGEN, Mexico City 14610, Mexico; daparicio@inmegen.gob.mx; 10 Department of Science and Engineering, University of Guanajuato, Leon 37670, Guanajuato, Mexico; gustavo.basurto@ugto.mx; 11 Laboratory of Fibrosis and Cancer, Faculty of Medicine and Surgery, ‘Benito Juárez’ Autonomous University of Oaxaca—UABJO, Oaxaca 68120, Mexico; rbaltierrezho@conacyt.mx (R.B.-H.); vrvasquezga@conacyt.mx (V.R.V.-G.); 12 Deputy Directorate of Humanistic and Scientific Research, National Council of Humanities, Sciences and Technologies—CONAHCYT, Mexico City 03940, Mexico; 13 Laboratory of Experimental Hepatology, Department of Pharmacology, Center for Research and Advanced Studies of the National Polytechnic Institute—CINVESTAV-IPN, Mexico City 07360, Mexico; pmuriel@cinvestav.mx

**Keywords:** alcoholic liver disease, cellular senescence, oxidative stress, protein carbonylation

## Abstract

Aging is characterized by increased reactive species, leading to redox imbalance, oxidative damage, and senescence. The adverse effects of alcohol consumption potentiate aging-associated alterations, promoting several diseases, including liver diseases. Nucleoredoxin (NXN) is a redox-sensitive enzyme that targets reactive oxygen species and regulates key cellular processes through redox protein–protein interactions. Here, we determine the effect of chronic alcohol consumption on NXN-dependent redox interactions in the liver of aged mice. We found that chronic alcohol consumption preferentially promotes the localization of NXN either into or alongside senescent cells, declines its interacting capability, and worsens the altered interaction ratio of NXN with FLII, MYD88, CAMK2A, and PFK1 proteins induced by aging. In addition, carbonylated protein and cell proliferation increased, and the ratios of collagen I and collagen III were inverted. Thus, we demonstrate an emerging phenomenon associated with altered redox homeostasis during aging, as shown by the declining capability of NXN to interact with partner proteins, which is enhanced by chronic alcohol consumption in the mouse liver. This evidence opens an attractive window to elucidate the consequences of both aging and chronic alcohol consumption on the downstream signaling pathways regulated by NXN-dependent redox-sensitive interactions.

## 1. Introduction

Aging is a natural process that causes progressive loss of tissue and organ function over time and is closely associated with systemic inflammation, among many other diseases [1]. Alcohol abuse is rapidly increasing among adults over 65 years old, a population more sensitive to the adverse effects of alcohol because oxidative stress and DNA damage resulting from alcohol misuse exacerbate age-associated diseases, such as liver diseases, and increase the risk of cancer [2].

Several well-known altered molecular mechanisms that trigger human aging, such as increased reactive oxygen species (ROS) production, inflammation, immune system decrease, and DNA damage response, among others, have been identified [3]. As a result, various tissues may enter into a permanent cell cycle arrest where both endogenous and exogenous stress response mechanisms contribute to this end throughout life [4]. Most cells cease to proliferate but remain metabolically active for a long period, called senescent cells [5]. It has been well established that the increased number of senescent cells in the liver strongly impacts its functioning and regeneration [6]. An increased rate of senescent cells decreases liver function during alcohol-related liver disease progression [4]. In addition, senescent cells secrete a large number of proinflammatory cytokines, chemokines, growth factors and proteases that may affect neighboring cells, a phenomenon known as the senescence-associated secretory phenotype (SASP) [7]. This phenomenon potentiates ROS production and oxidative DNA damage as ROS might attack nitrogenous bases, which are associated with premature aging when they are not completely repaired, and eventually promote malignancy [8].

The liver is a complex metabolic organ essential for maintaining whole-body homeostasis via regulation of energy metabolism, xenobiotic and endobiotic clearance, and molecular biosynthesis [9]. Thus, chronic liver insults might severely influence organ functioning, which increases with age [10]. Alcohol-related liver disease (ALD), a major cause of morbidity and mortality worldwide, encompasses a spectrum of altered hepatic manifestations ranging from simple steatosis to steatohepatitis, cirrhosis, and eventually hepatocellular carcinoma (HCC) [11]. One of the key players in ALD progression is oxidative stress since chronic alcohol consumption increases ROS, which induces lipid peroxidation and protein carbonylation, decreases the hepatic antioxidant defense, leading to hepatocellular damage [12], and promotes biomolecule modification, such as protein carbonylation [13]. Interestingly, although the activity of cytochrome P450-2E1, an enzyme involved in ethanol metabolism, decreases with age [14], it is implicated in promoting aging-related hepatic disease by increasing protein carbonylation and oxidative DNA damage, among other oxidation-associated damages [15].

Nucleoredoxin (NXN), a redox-sensitive and multifunctional enzyme, acts as an oxidoreductase by targeting ROS [16] and is necessary for the integrity of antioxidant systems [17]. It regulates different key cellular processes through redox-sensitive protein–protein interactions, including innate immunity, inflammation, proliferation, glycolysis, and neuronal plasticity, among others [18], which largely depend on the oxidative stress status of the cells [17]. Notably, some NXN interactions and their downstream signaling pathways are disrupted by ROS producers, such as H_2_O_2_ [19], acetaldehyde [20], and ethanol [21,22]. Moreover, NXN regulates signaling pathways that have been linked to the establishment of cellular senescence, such as the Wingless (WNT)/β-catenin and Toll-like receptor 4 (TLR4)/myeloid differentiation primary response 88 (MYD88) pathways [23,24]. Of interest, the impairment of redox homeostasis in aging may cause covalent modifications to deacetylases, which modulate signaling pathways such as WNT/β-catenin [25]. Therefore, an unexplored area is whether NXN-dependent redox-sensitive interactions, which might be targeted by oxidative stress induced by both aging and chronic alcohol consumption, are sensitized by aging and enhanced by chronic alcohol consumption in the mouse liver. Thus, using an ALD in vivo model [26], in this investigation we determine the effect of chronic alcohol consumption on NXN-dependent redox interactions in the liver of aged mice.

## 2. Materials and Methods

### 2.1. Experimental Design

**Figure 1:**
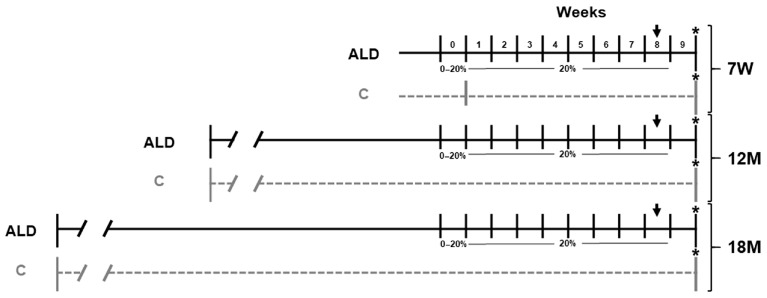
Schematic representation of ALD model in young and aged mice. Animal groups represented by continuous black lines received 20% (*v*/*v*) ethanol for 8 weeks, after which they received a single dose of LPS (1 mg/kg, i.p.) in the last week, which is indicated by a black arrow. Animal groups represented by dashed gray lines did not receive either ethanol or LPS. All animals were euthanized one week after the end of alcohol consumption, indicated by an asterisk (*). *n* = 5 animals/group. ALD, alcohol liver disease model; C, control; 7W, 7-week-old mice; 12M, 12-month-old mice; 18M, 18-month-old mice.

Female C57BL/6J mice were obtained from the Animal Production and Experimentation Unit of the Center for Research and Advanced Studies of the National Polytechnic Institute (UPEAL-CINVESTAV-IPN; Mexico City, Mexico) and were fed ad libitum and housed in a controlled environment with a 12 h light/dark cycle. Animal experimentation was following the Institutional Animal Use and Care Committee of CINVESTAV-IPN with the approved protocol No. 0114-14. To study ALD, animals were subjected to a protocol of chronic ethanol consumption, as previously reported [26]. Three groups (*n* = 5 per group) of animals aged 7 weeks, and 12 and 18 months, were first subjected to a six-day ramp-up period divided into two days per ethanol dosage, i.e., 5% (*v*/*v*) was increased per dose until reaching 20% (*v*/*v*) ethanol in 20% (*w*/*v*) sucrose. Then, animals were kept at 20% ethanol ad libitum for 8 weeks as the only source of drinking fluid (Figure 1). In the last week of ethanol consumption, animals received a single dose (1 mg/kg, i.p.) of lipopolysaccharide (LPS). The LPS dose was selected and used based on previous studies in which the acute effects of LPS in ALD models were evaluated [21,22]. Simultaneously, three groups of matched control animals received tap water and a standard diet ad libitum. Finally, animals were euthanized by exsanguination under isoflurane anesthesia, and immediately, liver pieces were divided for protein isolation, and frozen in liquid nitrogen for cryopreservation, and then stored at −75 °C for further analysis. Other pieces were fixed in 4% formalin and embedded in paraffin for histological or immunostaining analyses. The animal body weights were recorded at the beginning and at the end of alcohol consumption, while the percentage of relative liver weight was recorded immediately after euthanasia (Table 1).

### 2.2. Histopathology and Immunodetection Analyses

Liver tissues fixed in 4% formalin were embedded in paraffin, cut into 3-μm-thick slices, deparaffinized and gradually rehydrated. For histological examination and fibrosis detection, sections were processed by standard hematoxylin and eosin (H&E) and Masson’s trichrome (M’sT) staining, respectively. 

For immunohistochemistry (IHC) and immunofluorescence (IF) analyses; after tissue rehydration, antigens were unmasked and processed according to the Mouse on Mouse HRP-Polymer Bundle kit (MM510; BioCare Medical. Concord, CA, USA). Next, for IHC analysis, primary antibodies against collagen 3 alpha 1 (COL3A1; 1:200; NB600-594SS, Novus Biologicals; Centennial, CO, USA), interleukin 6 (IL-6; 1:100; 21865-1-AP; Proteintech; Rosemont, IL, USA), KI67 and collagen 1 alpha 1 (KI67; 1:200 and COL1A1; 1:200; GTX16667 and GTX112731, respectively; GeneTex; Irvine, CA, USA), were incubated overnight at 4 °C in the blocking solution provided in the kit (MM510; BioCare Medical). After a standard staining protocol using the HRP/DAB Mouse/Rabbit ImmunoDetector Detection System (BSB 0005, BioSB; Santa Barbara, CA, USA), sections were lightly counterstained with hematoxylin, dehydrated, and mounted. The positive label in liver tissue was quantified in 20 randomly selected 20× fields per individual sample, totaling 100 images per treatment group in a total of 600 images using ImageJ 1.5 software. For IF analyses, anti-proliferating cell nuclear antigen (PCNA; 10205-2-AP; Proteintech; Rosemont, IL, USA) and anti-poly (ADP-ribose) (PAR; 1:200; ALX804220R100, Enzo. Farmingdale, NY, USA), were incubated overnight at 4 °C. Tissues were then incubated for 1 h at room temperature (RT) in the dark with Alexa Fluor 488 anti-rabbit and anti-mouse antibodies (1:300; ab150077 and ab150117, respectively; Abcam; Cambridge, MA, USA). Nuclei were stained with DAPI (1:1000; 62247, Thermo Scientific; Rockford, IL, USA) for 5 min at RT. Finally, tissues were rinsed, drained, and fixed using Fluoroshield Mounting Medium (ab104135; Abcam; Cambridge, MA, USA). For quantifying the area and positive nuclei, 20 randomly selected 20× fields per individual sample were captured, totaling 100 images per treatment group, and 600 total images. The label was quantified using ImageJ 1.5 software. Images for IHC and IF analysis were captured using a ZEISS Axio-A1 microscope (Oberkochen, Germany).

Double-labeling for detecting NXN and phospho-H2A.X (p-H2A.X) was performed using the IF protocol on frozen tissue. Frozen 7-μm-thick liver sections were rinsed twice in ice-cold PBS, fixed with 3.7% formaldehyde in PBS (pH 7.4) for 10 min at RT, washed, and incubated for 50 min in washing solution [0.25% Triton X-100 in PBS (PBST)]. Sections were blocked with 1% bovine serum albumin (BSA) in PBST for 30 min at RT. Then, anti-p-H2A.X (Ser139) (1:50, 2577S; Cell Signaling; Danvers, MA, USA) antibody was incubated overnight at 4 °C in blocking solution. After washing, sections were incubated with Alexa Fluor 488 goat anti-rabbit (1:400, ab150077; Abcam; Cambridge, MA, USA) diluted in blocking solution for 1 h at RT in the dark. Then, anti-NXN (1:25, GTX107039; GeneTex; Irvine, CA, USA) antibody was incubated for 4 h at 4 °C in a blocking solution. After washing, sections were incubated with Alexa Fluor 568 goat anti-rabbit (1:1000, A-11079; Thermo Scientific; Rockford, IL, USA) diluted in blocking solution for 1 h at RT in the dark. Nuclear DNA was stained with DAPI (1:1000, 62247; Thermo Scientific; Rockford, IL, USA) for 5 min at RT. Finally, tissues were rinsed, drained, and fixed using Fluoroshield Mounting Medium (ab104137; Abcam; Cambridge, MA, USA). Pictures were captured using a Leica TCS SP8 AOBS (Acousto-optical Beam Splitter) DMI6000 confocal microscope (Wetzlar, Germany), provided by the Confocal Microscopy Unit at the Cell Biology Department of CINVESTAV-IPN (CONAHCYT–Mexico, Mexico City).

### 2.3. Senescence-Associated β-Galactosidase Activity In Situ (SA-β-gal)

SA-β-gal detection was performed, as previously described [27]. Briefly, frozen 10-μm thick liver sections were mounted on glass slides, fixed with fixing solution (1 mM MgCl_2_, 0.1 M EGTA, and 0.2% glutaraldehyde) for 5 min, washed with deionized water and incubated at 37 °C for 24 h with β-gal staining solution (0.4 M citric acid/sodium phosphate buffer, pH 6), 5 mM potassium ferrocyanide, 5 mM potassium ferricyanide, 1 M MgCl_2_, and 1 mg/mL X-gal). Sections were then counterstained with hematoxylin and dehydrated, and SA-β-gal-positive cells were captured accounting for 20 randomly selected either 40 or 100× fields per individual sample, i.e., 100 images per treatment group, and 600 total images. Images were captured using a ZEISS Axio-A1 microscope (Oberkochen, Germany).

To detect SA-β-gal activity alongside NXN protein in the same tissue section, frozen sections were treated for β-gal activity detection as previously described [27]. Briefly, after incubation with β-gal staining solution, sections were blocked with 1% BSA in PBS for 2 h and incubated for 1 h at 4 °C with anti-NXN (1:150; GTX107039, GeneTex; Irvine, CA, USA) antibody. Endogenous peroxidase activity was blocked for 12 min at RT; then, ImmunoDetector Biotinylated Link was added for 10 min, and then secondary InmunoDetector HRP-label conjugated antibodies and the DAB-Plus Substrate Kit were used for signal detection (BSB 0005, BioSB; Santa Barbara, CA, USA). Sections were then counterstained with hematoxylin and dehydrated, and SA-β-gal-positive cells were captured from 10 randomly selected 20, 40 and 100× fields per individual sample, totaling 50 images per treatment group, and 300 total images. Images were captured using a ZEISS Axio-A1 microscope (Oberkochen, Germany).

### 2.4. Western Blot (WB) Analysis

Total protein extracts were prepared in a lysis buffer as previously described [21]. Lysates were incubated for 30 min under shaking and centrifuged at 16,000× *g* for 10 min at 4 °C, and supernatants were stored at −70 °C for further analyses. After protein quantification, equivalent amounts of proteins were analyzed in Laemmli buffer by SDS–PAGE and transferred to a PVDF membrane. The antibodies used were anti-pH2A.X (2577S; Cell Signaling; Danvers, MA, USA), anti-NXN (GTX107039, GeneTex; Irvine, CA, USA), and anti-GAPDH (60004-1-Ig; Proteintech; Rosemont, IL, USA). Protein loading was confirmed by analyzing the blots with anti-GAPDH. Densitometric analyses were performed using ImageJ 1.5.29 software.

### 2.5. Determination of Carbonylated Proteins

The carbonyl content of proteins was detected using the OxyBlot protein oxidation detection kit (S7150; Sigma-Aldrich, Purchase, NY, USA) according to the manufacturer’s instructions, with minor modifications. To derivatize carbonyl groups for WB analysis, equal volumes of tissue lysate (10 mg/mL) were mixed in 12% SDS, followed by 2 volumes of 20 mM 2,4-dinitrophenylhydrazine in 10% trifluoroacetic acid. The mixture was incubated in the dark at RT for 20 min and the OxyBlot neutralization solution was added to stop the reaction; a nonderivative control per group was included. The primary antibody was an anti-rabbit polyclonal against dinitrophenylhydrazone, and the HRP-conjugated secondary antibody was used at 1:300 dilution. Bands revealed in the full lanes were quantified by densitometry analysis using the ImageJ 1.5.29 software.

### 2.6. Immunoprecipitation (IP) Assay

Total proteins from liver tissues were processed in a lysis buffer containing 20 mmol/L Tris-Cl, pH 7.8, 137 mmol/L NaCl, 1% (*v*/*v*) Nonidet P40, 10% (*v*/*v*) glycerol, and 2 mmol/L EDTA (all from Sigma; St. Louis, MO, USA), and supplied with Complete and PhoStop (Roche; Basel, Switzerland). Proteins of interest were immunoprecipitated with a mixture of protein A/G Plus-Agarose complex beads (sc2003, Santa Cruz Biotechnology; Santa Cruz, CA, USA) and rabbit anti-NXN (GTX107039; GeneTex; Irvine, CA, USA). Precipitated and coprecipitated proteins were analyzed by WB and normalized by reanalyzing the blots for the constant regions (heavy chains) of the immunoglobulins used for IP assays.

To detect precipitated and coprecipitated proteins, anti-NXN (AF-5718, Novus Biologicals; Centennial, CO, USA), anti-flightless (FLII) and anti-phosphofructokinase 1 (PFK1) (sc-21716 and sc-166722, respectively, Santa Cruz Biotechnology; Santa Cruz, CA, USA), anti-MYD88 (4283S, Cell Signaling; Danvers, MA, USA), and anti-calcium/calmodulin-dependent protein kinase II type alpha (CAMK2A) (SAB4503250, Merck; Darmstadt, Germany) antibodies were used. To avoid cross-reactivity with the denatured heavy or light chains of the antibodies used for IP assays, goat anti-mouse and mouse anti-rabbit light chain-specific secondary antibodies (115-035-174 and 211-032-171, respectively; Jackson ImmunoResearch, Baltimore, PA, USA) were used.

### 2.7. Statistical Analysis

Statistical analyses were performed using GraphPad Prism 8.0 software (GraphPad, La Jolla, CA, USA). Five animal per group were included and data calculation was performed using a one-way ANOVA test for multiple comparisons. Data are expressed as the mean ± SE. Differences were considered significant when *p* < 0.05.

## 3. Results

### 3.1. Chronic Alcohol Consumption Increases Mice Body Weight and the Relative Liver Weight during Aging

Although aged mice subjected to the ALD model recorded higher body weights (both *p* < 0.0001) at the end of ethanol consumption, i.e., 12- and 18-month-old mice, as compared with the onset of treatment, that of control groups were also significantly increased (both *p* < 0.0001). Body weight of young animals was not different at the end of the experiment. Interestingly, the percentage of the relative liver weight was only increased in 18-month-old mice subjected to the ALD model (*p* < 0.0001) as compared with its respective control (Table 1).

**Table 1:** Effect of chronic ethanol consumption on mice body weight and relative liver weight.

Group	Age	Body Weight (g)		Percentage of Relative Liver Weight
		Initial	Final	
C	7W	15.28 ± 0.16	15.64 ± 0.14	5.53 ± 0.12
	12M	24.93 ± 2.60	29.92 ± 1.39 *	5.65 ± 0.66
	18M	32.00 ± 2.08	36.00 ± 1.05 *	4.38 ± 0.28
ALD	7W	17.66 ± 0.37	18.44 ± 0.30	4.97 ± 0.16
	12M	26.40 ± 0.58	30.34 ± 0.81 *	6.14 ± 0.31
	18M	26.92 ± 0.68	30.30 ± 0.47 *	6.25 ± 0.64 ^$^

Values represent the average ± SE. * Statistically different from the initial body weight; ^$^ Statistically different from the respective control group; *p* < 0.0001.

### 3.2. Chronic Alcohol Consumption Modifies Extracellular Matrix Components during Aging

**Figure 2:**
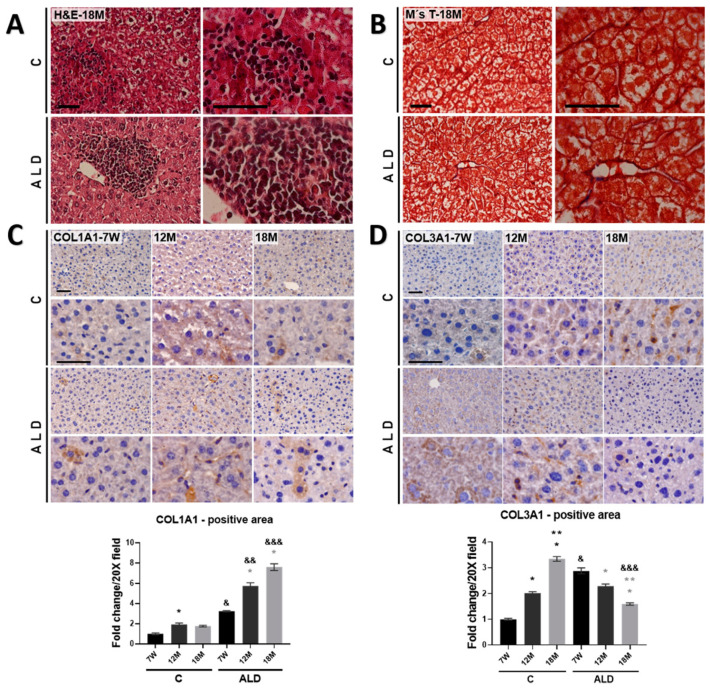
Extracellular matrix components determination in the liver of aged mice. (**A**) H&E staining. (**B**) M’sT staining. Magnification is 40 and 100× and the scale bar indicates 50 μm. (**C**,**D**) IHC analysis and signal quantification of COL1A1 and COL3A1 proteins. Magnification is 40 and 100× and the scale bar indicates 50 μm. Bars represent mean ± SE. * Significantly different from 7W, ** from 12M compared to the C group, * from 7W, ** from 12M compared to the ALD group, ^&^ from 7W, ^&&^ from 12M, ^&&&^ from 18M compared to the respective control group; *p* < 0.05. *n* = 5 animals/group. ALD, alcohol liver disease model; C, control; 7W, 7-week-old mice; 12M, 12-month-old mice; 18M, 18-month-old mice.

Histopathological analysis by H&E staining showed a mild inflammatory infiltrate alongside disarrangement of the hepatocyte cords in 18-month-old mice of both control and ALD groups (Figure 2A). As shown in Figure 2B, M’sT staining revealed a slight signal of collagen fibers in the liver tissues of 18-month-old mice subjected to the ALD model. Then, by IHC analysis, we analyzed COL1A1 and COL3A1 levels, two key components of the extracellular matrix (ECM) altered during the progression of liver fibrosis [28]. As shown in Figure 2C, the level of COL1A1 was increased (*p* = 0.0158) in 12-month-old control mice as compared with that in young control mice, i.e., 7-week-old mice; notably, in the ALD model, COL1A1 was increased in 12- and 18-month-old mice (both *p* < 0.0001). Contrastingly, the level of COL3A1 in 12- and 18-month-old control mice was significantly (both *p* < 0.0001) increased, but in the ALD model, COL3A1 was decreased (*p* < 0.0001) in both 12- and 18-month-old mice as compared with young mice subjected to the ALD model.

### 3.3. Cell Proliferation Is Increased by Chronic Alcohol Consumption during Aging

**Figure 3:**
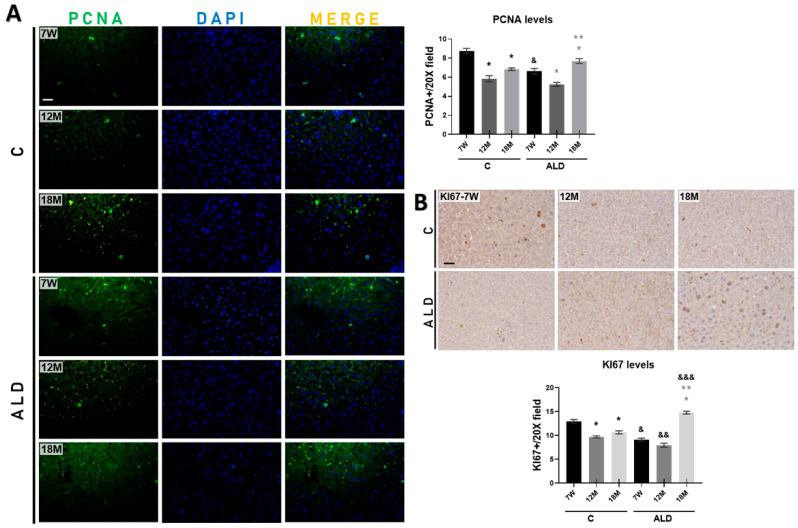
Effect of chronic alcohol consumption on cell proliferation in liver tissue of young and aged mice. (**A**) IF analysis and signal quantification of PCNA protein. Magnification is 20× and the scale bar indicates 100 μm. (**B**) IHC analysis and signal quantification of KI67 protein. Magnification is 20× and the scale bar indicates 100 μm. Bars represent the mean ± SE. * Significantly different from 7W, * from 7W, ** from 12M compared to the ALD group, ^&^ from 7W, ^&&^ from 12M, ^&&&^ from 18M compared to the respective control group; *p* < 0.05. *n* = 5 animals/group. ALD, alcohol liver disease model; C, control; 7W, 7-week-old mice; 12M, 12-month-old mice; 18M, 18-month-old mice.

The evidence has shown that the regenerative capability of the liver decreases with age [29]. Thus, to determine the effect of chronic alcohol consumption on aged livers, we measured the proliferation markers PCNA and KI67 levels. IF analysis revealed that PCNA-positive cells were decreased in both 12- and 18-month-old control mice (both *p* < 0.0001) as compared to young control animals; however, the ALD model increased PCNA-positive cells in 18-month-old mice (*p* = 0.0472), but they were decreased in 12-month-old mice (*p* = 0.0023) as compared with young animals. PCNA-positive cells were decreased in 7-week-old mice subjected to the ALD model (*p* < 0.0001) as compared with the respective young controls (Figure 3A). IHC analysis revealed that the number of KI67-positive cells in 12- and 18-month-old control mice decreased (both *p* < 0.0001) as compared with that in young control animals, while in the ALD model, this number increased (*p* < 0.0001) in 18-month-old mice as compared with that in young animals. Interestingly, young and 12-month-old mice subjected to the ALD model showed a reduced number of KI67-positive cells (*p* < 0.0001 and *p* = 0.0039, respectively) as compared with their respective untreated controls (Figure 3B).

### 3.4. Chronic Alcohol Consumption Increases Cellular Senescence Markers in the Liver of Aged Mice

**Figure 4:**
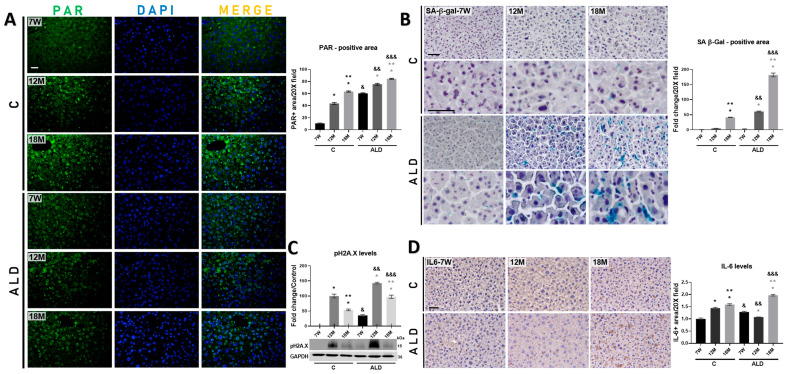
Determination of DNA damage and cellular senescence markers induced by chronic alcohol consumption in the liver of young and aged mice. (**A**) IF analysis and signal quantification of PAR protein. Magnification is 20× and the scale bar indicates 100 μm. (**B**) Histochemical analysis and signal quantification of SA-β-gal activity. Magnification is 20 and 40×, and the scale bar indicates 50 μm. (**C**) WB analysis and intensity quantification of pH2A.X protein. pH2A.X protein levels were normalized with those of GAPDH protein, which was used as a loading control. (**D**) IHC analysis and signal quantification of IL-6 protein. Magnification is 20× and the scale bar indicates 50 μm. Bars represent the mean ± SE. * Significantly different from 7W, ** from 12M compared to the C group, * from 7W, ** from 12M compared to the ALD group, ^&^ from 7W, ^&&^ from 12M, ^&&&^ from 18M compared to the respective control group; *p* < 0.05. *n* = 5 animals/group. ALD, alcohol liver disease model; C, control; 7W, 7-week-old mice; 12M, 12-month-old mice; 18M, 18-month-old mice.

An early event in the double-strand DNA break (DSB) response is the rapid recruitment and activation of PARP1, resulting in PAR polymerization, an early marker of DNA damage, followed by histone recruitment to DNA damage sites to stimulate chromatin remodeling and DNA repair [30]. To determine the status of DNA damage associated with aging and/or promoted by chronic alcohol consumption, we quantified PAR-positive cells by IF analysis. Results showed that PAR increased in both 12- and 18-month-old control mice livers (both *p* < 0.0001) as compared with young animals. Of note, the ALD model induced PAR levels in the livers of both 12- and 18-month-old mice (both *p* < 0.0001) as compared with young animals, but the ALD model also strongly induced PAR levels (*p* < 0.0001) in young mice as compared with young control animals, as well as in both 12- and 18-month-old mice livers (both *p* < 0.0001) as compared with their respective untreated controls (Figure 4A).

Since there is no single unequivocal marker of cellular senescence, we determined the presence of several well-known markers associated with this phenomenon, such as SA-β-gal activity, pH2A.X, and IL-6 [7]. As expected, histochemical analysis revealed that the activity of the SA-β-gal enzyme increased in the livers of 18 month-aged control mice as compared with 12-month-old and young animals; interestingly, SA-β-gal activity was strongly induced in the livers of both 12- and 18-month-old mice subjected to the ALD model as compared with young animals and with their respective untreated controls (Figure 4B). WB analysis revealed that pH2A.X levels increased in both 12- and 18-month-old control mice (both *p* < 0.0001); however, as shown in Figure 4C, chronic alcohol consumption induced the level of this protein in all animal groups subjected to this scheme, namely 7-week-old, 12- and 18-month-old mice (all *p* < 0.0001). In addition, IHC analysis demonstrated that the IL-6-positive area increased in the livers of 12- and 18-month-old control mice (both *p* < 0.0001) compared with those of young mice. The ALD model increased its level only in 18-month-old mice (*p* < 0.0001) as compared with 7-week-old and 12 month-aged mice, as well as with its respective untreated control (Figure 4D).

### 3.5. Chronic Alcohol Consumption Promotes the Preferential Localization of NXN Either into or Alongside Senescent Cells in the Liver of Aged Mice

**Figure 5:**
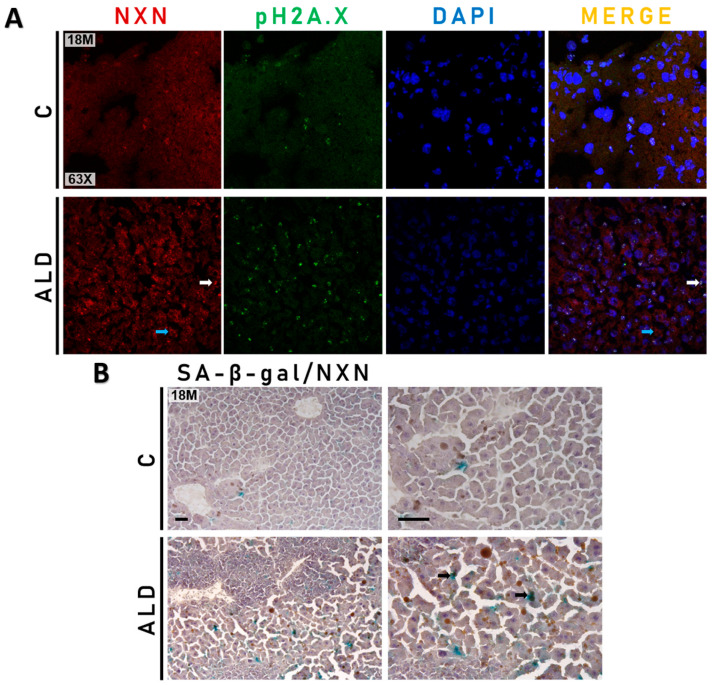
Co-labeling of NXN with pH2A.X and SA-β-gal activity in the liver of aged mice (**A**) Double staining of NXN and pH2A.X proteins by IF analysis. White and blue arrows point out nuclear and cytoplasmic localization of NXN, respectively. Magnification is 63×. (**B**) Double staining of SA-β-gal activity and NXN protein by IHC and histochemical analyses, respectively. Black arrows indicate double staining of SA-β-gal activity and NXN. Magnification is 20 and 40× and the scale bar indicates 50 μm. Pictures show tissues from 18-month-old mice. ALD, alcohol liver disease model; C, control.

Based on the increased activity of the SA-β-gal enzyme in the liver of 18-month-old mice (Figure 4B), we then determined whether there was a possible relationship between senescence markers and NXN in the liver tissue of aged mice. Double staining of NXN with pH2A.X protein and SA-β-gal activity by IHC and IF analyses, respectively, in the liver of 18-month-old mice revealed that NXN was preferentially localized in the nucleus and cytoplasm of either senescent or adjacent cells in animals subjected to the ALD model (Figure 5A,B).

### 3.6. Carbonylated Proteins Are Increased by Chronic Alcohol Consumption in the Liver of Aged Mice

**Figure 6:**
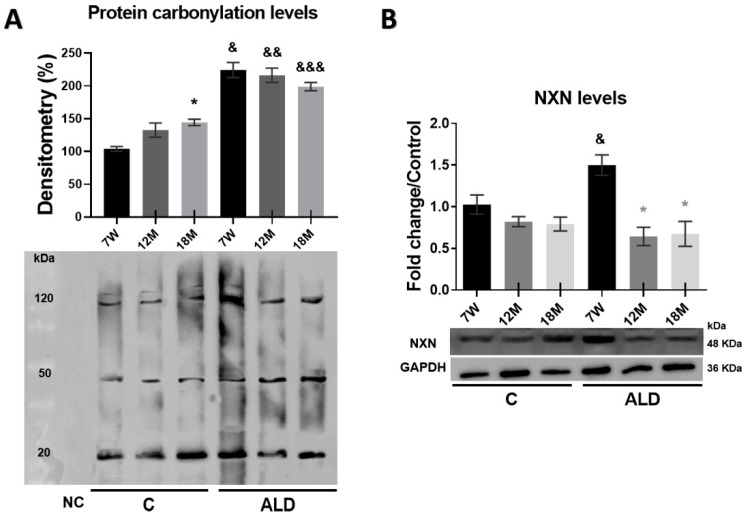
Effect of chronic alcohol consumption on protein carbonylation and NXN protein levels in the liver of young and aged mice. Two pools of three individual samples each were made by mixing equal amounts of proteins. (**A**) Determination of carbonylated proteins by OxyBlot analysis. (**B**) WB analysis of NXN protein. NXN protein level was normalized with that of GAPDH, which was used as a loading control. Bars represent the mean ± SE. * Significantly different from 7W, * from 7W, ^&^ from 7W, ^&&^ from 12M, ^&&&^ from 18M compared to the respective control group; *p* < 0.05. *n* = 5 animals/group. ALD, alcohol liver disease model; C, control; 7W, 7-week-old mice; 12M, 12-month-old mice; 18M, 18-month-old mice.

Among a wide range of ROS-derived modifications, carbonylation of biomolecules is a major hallmark of oxidative stress and determines the oxidation degree associated with cellular damage, aging, and several age-related disorders [31]. Carbonylated proteins analysis showed that the baseline observed in young control animals was increased (*p* = 0.0197) in 18-month-old control mice; interestingly, chronic alcohol consumption promoted the level of carbonylated proteins in the liver of all animal groups of the ALD model, namely 7-week-old, 12- and 18-month-old mice (*p* < 0.0001, *p* < 0.0001 and *p* = 0.0006, respectively) as compared with their respective untreated controls (Figure 6A). The measurement of NXN protein level revealed that while no changes were observed among control groups, chronic alcohol consumption stimulated its level in 7-week-old mice subjected to the ALD model (*p* = 0.0047); this phenomenon was not observed in both 12- and 18-month-old mice (Figure 6B).

### 3.7. Chronic Alcohol Consumption Worsens Aging-Promoted Alteration of NXN-Dependent Interaction Ratios in the Mouse Liver

**Figure 7:**
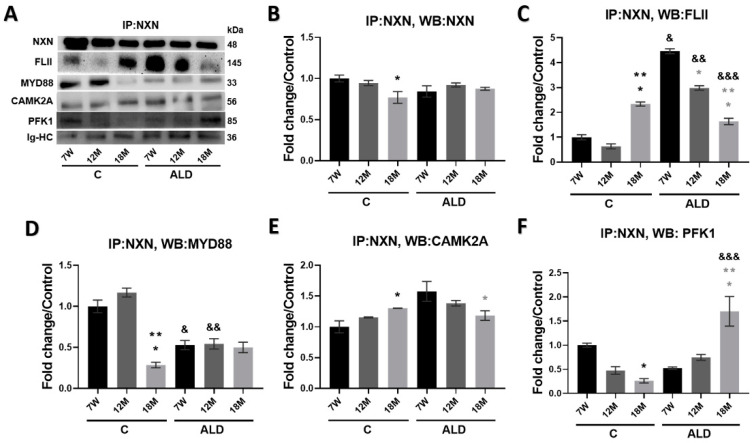
Effect of chronic alcohol consumption on NXN/FLII, NXN/MYD88, NXN/CAMK2A and NXN/PFK1 interaction in the liver of young and aged mice. Total protein extracted from mice was incubated to precipitate NXN in complex with its interacting proteins by using an anti-NXN antibody as indicated in materials and methods section. Two pools of three individual samples each were made by mixing equal amounts of proteins. (**A**) WB analyses were used to detect immunoprecipitated NXN and co-precipitated FLII, MYD88, CAMK2A and PFK1 proteins. Densitometry quantification of (**B**) NXN; (**C**) FLII; (**D**) MYD88; (**E**) CAMK2A and (**F**) PFK1 protein intensity. Immunoglobulin heavy chain (Ig-HC) bands, used for IP assay (anti-NXN), were detected to normalize protein levels. Bars represent the mean ± SE. * Significantly different from 7W, ** from 12M compared to the C group, * from 7W, ** from 12M compared to the ALD group, ^&^ from 7W, ^&&^ from 12M, ^&&&^ from 18M compared to the respective control group; *p* < 0.05. *n* = 5 animals/group. ALD, alcohol liver disease model; C, control; 7W, 7-week-old mice; 12M, 12-month-old mice; 18M, 18-month-old mice.

It has been well established that NXN interacts, in a redox-dependent manner, with several proteins such as FLII, MYD88, CAMK2A, and PFK1, among others, to regulate the downstream activity of different signaling pathways and, as a consequence, contributes to cellular redox homeostasis [18]; furthermore, some of the NXN redox-sensitive interactions are affected by chronic alcohol consumption [21,32]. Thus, to determine whether aging modifies the ratio of NXN/FLII, NXN/MYD88, NXN/CAMK2A, and NXN/PFK1 redox-sensitive interactions and whether chronic alcohol consumption worsens the aging-induced alterations in the liver, we performed IP analyses from total protein extracts. Results demonstrated that NXN was precipitated (Figure 7A,B), and FLII, MYD88, CAMK2A, and PFK1 were efficiently coprecipitated by an anti-NXN antibody (Figure 7A). Of note, in 18-month-old control mice, the NXN/FLII interaction ratio was strongly (*p* < 0.0001) increased, but the strongest increment of this interaction ratio was observed in the 7-week-old mice subjected to the ALD model, which was gradually decreased in both 12- and 18-month-old mice (both *p* < 0.0001). Notably, in 18-month-old mice subjected to chronic alcohol consumption, this interaction ratio was also decreased (*p* < 0.0001) as compared with its respective untreated control (Figure 7A,C). The NXN/MYD88 interaction ratio was found to be strongly decreased (*p* < 0.0001) in 18-month-old control mice as compared with both young and 12-month-old control mice; however, it was similarly reduced in young and aged mice subjected to the ALD model, even below the interaction ratio observed in their respective controls, especially in 7-week-old and 12-month-old mice (*p* = 0.0001 and *p* < 0.0001, respectively) as compared with their respective untreated control mice (Figure 7A,D). On the other hand, the interaction ratio of the NXN/CAMK2A complex was induced in 7-week-old mice (*p* = 0.0018) of the ALD model as compared with its untreated control, but it gradually decreased, reaching significance in 18-month-old mice (*p* = 0.0426) as compared with 7-week-old mice. Untreated controls showed a non-significant increasing trend during aging (Figure 7A,E). Finally, the interaction ratio of the NXN/PFK1 complex in untreated animals showed a gradual and significant (*p* = 0.0121) decrease in 18-month-old mice as compared with the young animals; contrarily, this interaction complex was induced by chronic alcohol consumption in 18-month-old mice (*p* < 0.0001) as compared with its respective untreated control (*p* < 0.0001), as well as compared with 7-week-old and 12-month-old mice (*p* = 0.0001 and *p* = 0.0011, respectively) subjected to the ALD model (Figure 7A,F).

## 4. Discussion

Deterioration of liver functioning due to aging contributes to the progression of age-related liver disease [33]. A well-described mechanism is that during aging, hepatic stellate cells adopt aging-related changes and secrete various inflammatory and tumor-promoting factors, including IL-1β, IL-6 and CXCL7, to induce neutrophil infiltration. Then, neutrophil-derived excessive oxidative stress induces DSB and restricts the proliferation of liver progenitor cells, leading to the impairment of liver regeneration [34]. Accumulating evidence indicates that an aged liver is more susceptible to injury due to alcohol abuse [35], which exacerbates oxidative stress and unbalances the redox status, playing a critical role in the alcohol-mediated cellular fate [36]. Since NXN is a redox-sensitive enzyme that has been proposed as a master regulator of cellular redox homeostasis [18], here we determine the effect of chronic alcohol consumption on NXN-dependent redox interactions in the liver of aged mice and identify that some NXN redox interactions are sensitized by aging and worsened by chronic alcohol consumption, which might potentiate the liver malfunctioning as discussed below.

Following hepatic injury, hepatic stellate cells undergo a complex activation process and become the leading source for the increased and irregular deposition of ECM components, leading to fibrosis [37]. During a normal fibrillogenesis process, while the level of COL1A1 is decreased, that of COL3A1 is increased; however, in liver fibrotic tissues, this homeostatic collagen I/III ratio is inverted [28]. This altered ratio can be found in the liver of aged individuals and might be associated with a chronic low-grade proinflammatory state [38]. Although M’sT staining did not clearly detect deposition of collagen fibers, IHC analyses revealed that chronic alcohol consumption increased COL1A1 levels and decreased that COL3A1 levels in the livers of aged mice. This phenomenon was accompanied by a mild inflammatory infiltrate and disarrangement of the hepatocyte cords (Figure 2). Although collagen fibers were not detected by M’sT staining, a less sensitive procedure than immunodetection, this evidence indicates that both the reversal of the collagen I/III ratio and the slight inflammatory infiltrate were favored by aging in the liver of animals subjected to the ALD model, as some of the initial alterations might progress to a worse condition if the liver continues to be exposed to chronic insult by either a single or multiple promoting factors for a more extended time.

Cell proliferation is an essential process for the growth and maintenance of tissue homeostasis. Some widely used proliferation markers, such as PCNA and KI67 proteins, have been helpful for identifying alterations in the cell proliferation process [39]. Although the liver is an organ with high proliferative potential that promotes its regeneration after partial tissue removal or cellular damage, a phenomenon known as compensatory proliferation, its regenerative capability declines with aging [40]. It has been reported that chronic alcohol consumption for 24 months, increases liver cell proliferation from the onset up to 7 months of alcohol consumption; interestingly, at 12 months, it is decreased but at 18 months, the proliferation ratio is similar to controls [41]. As expected, our results show that KI67 and PCNA levels decreased in aged-untreated animals, i.e., 12- and 18-month-old mice; however, chronic alcohol consumption increased their protein levels in 18-month-old mice (Figure 3). Of note, this result is in concordance with the increased relative liver weight observed in the same animal group (Table 1). A plausible explanation for this contrasting result when compared with the previously reported data [41], is that the compensatory proliferation induced by the hepatotoxic effects of chronic alcohol consumption, is boosted in the initial times of alcohol exposure and afterward, the cells adapt to alcohol cytotoxicity, as occurs with exposure to high levels of LPS promoted by ethanol-induced intestinal barrier dysfunction [42]. This is because at the time of evaluation, in our ALD model, the animals had been subjected to alcohol consumption only for the first 8 weeks at 18-month-old, but in the study by Mendenhall and coworkers [41], the animals had been fed alcohol for 18 months at the same age. Thus, the induction of compensatory proliferation by alcohol might be associated with the onset of exposure of liver cells to chronic alcohol consumption.

Ethanol-induced oxidative stress might lead to cellular senescence by increasing ROS production and reducing the activity of the cellular antioxidant system, which increase DNA damage [12]. In response to DNA damage, DNA-dependent PARP, especially PARP-1, quickly recognizes both single- and DSB ends. Then, PAR is synthesized, a DNA damage signaling molecule that allows a rapid and efficient cellular evaluation of DNA damage and concentrates key factors of the single-strand break repair pathway at the damage site [30]. Interestingly, it has been reported that the inactivation of *Parp-1* gene in mice leads to acceleration of aging [43]. Our IF analysis revealed that PAR level was significantly increased in untreated aged mice but was exacerbated by chronic alcohol consumption in both young and aged mice (Figure 4A). In vitro assays have shown that senescence, a stress response that universally occurs within tissues under pathophysiological conditions, is stimulated in hepatocytes by ethanol, suggesting that it plays a key role in ALD [44]; moreover, in hepatocyte cultures exposed to ethanol the SA-β-gal activity, a widely used biomarker of cellular senescence, is increased [45]. Interestingly, our results showed that the activity of SA-β-gal was increased in untreated 18-month-old mice, but this increment was observed from 12 months on and potentiated in 18-month-old mice subjected to the ALD model (Figure 4B), indicating that chronic alcohol consumption exacerbated the appearance of senescent cells in the liver of aged mice. 

Telomere shortening is an important event in senescence, which correlates with senescence-associated heterochromatin foci, a facultative heterochromatin domain associated with irreversible cell cycle arrest [46]. pH2A.X, a replacement histone, is the second most common marker of cellular senescence after SA-β-gal activity and is the most sensitive marker of DSB and telomere shortening [47]. Our results demonstrated that pH2A.X level was induced in the livers of 12- and 18-month-old mice of both untreated and treated with ethanol, but it was higher in those that consumed ethanol (Figure 4C). Moreover, most senescent cells may exhibit a SASP and overproduce massive proinflammatory cytokines and growth factors. IL-6 is one of the basic proinflammatory cytokines in SASP components [48]. It has been reported that in the livers of aged rats, *Il-6* mRNA is increased mainly in Kupffer cells, suggesting that these cells might be the main source of this cytokine in aged livers [49]. In addition, when acute injury occurs, the liver loses hepatocellular functions and induces compensatory regeneration, and IL-6, along with growth factors, promotes hepatocyte proliferation by increasing both DNA synthesis and genomic instability [50]. Our results showed that chronic alcohol consumption increased the IL-6 level in aged mice (Figure 4D). Altogether, these data indicate that the DNA repair mechanism is activated by aging itself in an attempt to reverse aging-associated cell damage, but it is potentiated by the cytotoxic effect of chronic alcohol consumption as an early damage event in both aged and young liver cells; thus, this damage leads to the establishment of cellular senescence, which might contribute to accelerating ALD progression during aging.

Oxidation causes structural damage to macromolecules that accumulates with age and contributes to disruption in signaling pathways, inadvertently resulting in cellular aging. It has been shown that redox-regulated signaling networks control SASP, which plays an important role in driving age-related diseases [51]. NXN is a redox-sensitive enzyme that maintains redox homeostasis, regulates several signaling pathways, and targets ROS [18]. Thus, we determined the co-labeling of NXN with pH2A.X and SA-β-gal by IF and IHC analyses, respectively, to identify a possible relationship between cellular senescence and NXN, a redox-sensitive enzyme that has not been previously associated with this cellular process. Our results showed that NXN either co-localized with or was preferentially detected alongside senescent cells (Figure 5). Since NXN targets ROS [18], this evidence strongly suggests that NXN is localized within or near senescent cells to protect them from oxidative stress produced by the senescent cells themselves and/or by ethanol metabolism.

Protein carbonylation is an irreversible and irreparable oxidative post-translational modification that yields a reactive carbonyl moiety. It has been shown that protein carbonylation is associated with several human diseases, increases with age, and is linked to the age-dependent depletion of specific enzymes [52]. Notably, protein carbonylation is increased in the liver of young mice exposed to chronic alcohol consumption for four months [53]. We observed that protein carbonylation was increased in untreated 18-month-old mice, but alcohol consumption administered for only eight weeks increased the level of carbonylated proteins in the liver of both young and aged mice, indicating that protein carbonylation is an early oxidative modification induced by ethanol, and aging contributed to potentiating this phenomenon (Figure 6A). A plausible mechanism in aged livers is that increased ROS production by ethanol consumption impairs mitochondria structure and function and increases the content of carbonylated proteins in mitochondrial but not those in the cell cytosol, as previously reported [12]. On the other hand, although NXN has not been implicated in cellular senescence, it may contribute based on its role in ROS and cellular redox homeostasis regulation [18]. Our results showed that NXN protein levels were strongly induced in the livers of young mice that consumed alcohol but not in aged mice (Figure 6B). A plausible explanation for this phenomenon is that in young animals, NXN still has the full capability to fight the increased oxidative stress and reverse the oxidative damage induced by alcohol. However, the enzymatic activity of NXN had already declined in aged mice, as occurs with alcohol-metabolizing liver enzymes in elderly people [15]. This decline might result from the oxidative modification of the NXN enzyme promoted by the carbonylation induced by chronic alcohol consumption, an intriguing hypothesis that needs to be addressed.

Nucleoredoxin interacts in a redox-dependent manner with seven proteins so far, regulates several cellular processes, and has been associated with different pathologies [18]. As a first approach to elucidating whether its protein–protein interaction ratios are modified with aging, we evaluated the interaction status of NXN with FLII, MYD88, CAMK2A and PFK1 proteins, which regulate innate immunity, inflammation, neuronal plasticity and glycolysis, respectively. While MYD88 is considered a hub of the inflammatory signaling pathways downstream of TLRs and other receptors [54], FLII, which also interacts with MYD88, is a multifunctional protein and has recently been identified as an emerging regulator of inflammation [55]. During a proinflammatory stimulus, MYD88 is recruited to TLR4, leading to the activation of NF-κB to transcribe genes involved in innate immunity and inflammation. To avoid unnecessary hyperactivation of the TLR4/MYD88 pathway, FLII hijacks MYD88 from TLR4 through NXN, forming the FLII/NXN/MYD88 complex [56]. We found that FLII increased its interaction ratio with NXN in the liver of young mice, but this interaction was strongly decreased in those aged mice subjected to the ALD model, while the MYD88 interaction ratio was unchanged in these animal groups. Of note, in untreated 18-month-old mice, the FLII/NXN interaction ratio was increased (Figure 7C,D). This evidence strongly suggests that chronic alcohol consumption decreases NXN’s efficiency to interact with FLII, likely due to the increased protein carbonylation induced by chronic alcohol consumption, which might be affecting redox-sensitive proteins such as NXN, a phenomenon that might be sensitized by aging but exacerbated by chronic alcohol consumption. 

Recently, it was reported that NXN interacts with and oxidizes CAMK2A to maintain its oxidative status and activity in neurons [57]. CAMK2A, a serine/threonine kinase, is highly abundant in the brain and plays several roles mediated by the regulation of intracellular Ca^2+^, such as neurotransmitter synthesis, ion channel regulation, cell division, muscle contractility, and gene transcription [18,57]. To our knowledge, its role in the liver has not been investigated. We observed that the NXN/CAMK2A interaction ratio decreased in aged mice subjected to the ALD model (Figure 7E), which might be a consequence of the impaired oxidase capability of NXN initiated by aging and increased by chronic alcohol consumption, affecting CAMK2A-dependent roles in the liver that remain to be investigated.

Phosphofructokinase-1 is a crucial regulator enzyme of glycolysis associated with the promotion of cancer cell proliferation since it is elevated in cancer tissues [58]. It has been shown that NXN interacts with PFK1 and stabilizes its oligomerization to regulate its catalytic activity. Because NXN deficiency increases NADPH and reduces glutathione levels, two major cellular antioxidants generated through the pentose phosphate pathway, it has been proposed that PFK1 makes cells more resistant to oxidative stress. Although it is still unknown how NXN might stabilize PFK1 oligomerization, it has been proposed that there might be a joint effect among NXN activity, glycosylation, phosphorylation and acylation since PFK1 is also subject to these post-translational modifications [59]. We found that the NXN/PFK1 interaction ratio decreased in untreated aged mice but increased in those subjected to chronic alcohol consumption (Figure 7F). Thus, this evidence suggests that a mechanism by which chronic alcohol consumption during aging increases cell proliferation (Figure 3) is through PFK1 stabilization by the contribution of NXN as well as some post-translational modifications. Further analysis will confirm this proposal.

## 5. Conclusions

**Figure 8:**
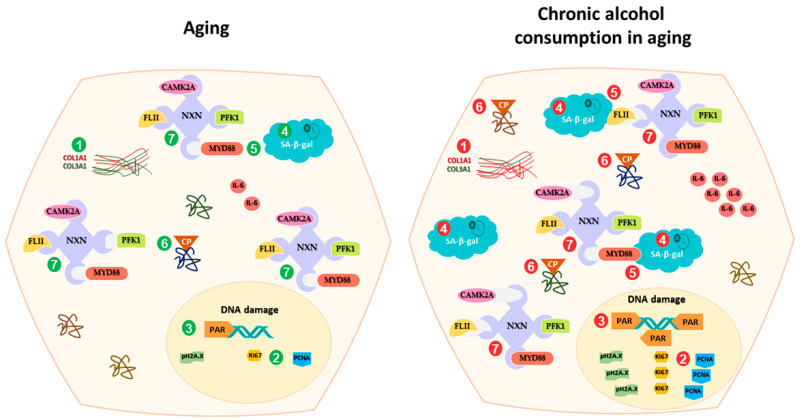
Schematic summary of chronic alcohol consumption effects on NXN-dependent redox-sensitive interactions during aging. Chronic alcohol consumption worsens the adverse effects of aging on (1) COL1A1/COL3A1 ratio, (2) cell proliferation, (3) DNA damage, (4) SA-β-gal activity, (5) NXN protein level and localization, (6) Protein carbonylation, and (7) the interaction of NXN with FLII, MYD88, CAMK2A and PFK1 proteins in the mouse liver. CAMK2A, calcium/calmodulin-dependent protein kinase II type alpha; COL1A1, collagen 1 alpha 1; COL3A1, collagen 3 alpha 1; CP, carbonylated protein; FLII, flightless; IL-6, interleukin 6; MYD88, myeloid differentiation primary response 88; NXN, nucleoredoxin; PAR, poly (ADP-ribose); PCNA, proliferating cell nuclear antigen; PFK1, phosphofructokinase 1; pH2AX, phospho-H2A.X (Ser139); SA-β-gal, senescence-associated β-galactosidase activity.

We provide evidence showing that chronic alcohol consumption worsens the alteration of NXN-dependent redox-sensitive interactions promoted by aging in the mouse liver, such as that of NXN with FLII, MYD88, CAMK2A and PFK1 proteins. Although NXN was preferentially localized either into or alongside senescent cells, which could be an attempt to protect the liver tissue from the adverse effects of ethanol, its interacting capability had already declined with aging. This was accompanied by an increase in SA-β-gal activity, PAR, pH2A.X, and IL-6 levels, protein carbonylation and cell proliferation, as well as the modification of the collagen I and collagen III ratios (Figure 8). Moreover, given that this evidence, to our knowledge, is the first to show the involvement of NXN in aging, our finding opens a window to escalate research addressed in elucidating how deeply the NXN interactions and their downstream signaling pathways are involved in aging and in the challenges that the liver faces when exposed to toxic agents, such as ethanol. Particularly, as it is well-known in the general population, ethanol intake is an independent predictor of other liver diseases, such as cirrhosis and HCC in patients bearing chronic hepatitis C virus infection, and of death in patients bearing either hepatitis C or B virus infection [60]. Thus, it is plausible that the disruption of NXN interactions can be potentiated by the concomitant effects of ethanol chronic intake and hepatitis virus infection. Although this proposal was not addressed and might be a limitation of our research, it is also an encouraging hypothesis arising from the current findings. Thus, the involvement of NXN in aging represents an emerging and attractive phenomenon that should continue to be investigated.

## Data Availability

Data are contained within the article.
